# International expert consensus on transdermal drug delivery technologies in dermatology

**DOI:** 10.1016/j.jdin.2026.04.024

**Published:** 2026-06-19

**Authors:** Jinjin Zhu, Yan Li, Xiuli Wang, Leihong Xiang, Martin Röcken, Vincent Piguet, Shyam Verma, Diamant Thaçi, Chunying Li, Xiang Chen, Mingji Zhu, Xiaoping Miao, Honglin Wang, Cheng Zhou, Yan Ding, Jun Xie, Yuling Shi, Mitchel P. Goldman, Fei Hao, Qiri Mu, Xiaoyong Man, Wen-Hung Chung, Xue Wang, Rong Xiao, Nan Yu, Aijun Chen, Jintao Zhu, Yong Cui, Lin Ma, Ping Yin, Wenbo Bu, Liu Yang, Jun Li, Xinghua Gao, Juan Tao

**Affiliations:** a Department of Dermatology, Union Hospital, Tongji Medical College, Huazhong University of Science and Technology, Wuhan, China; b Institute of Photomedicine, Shanghai Skin Disease Hospital, School of Medicine, Tongji University, Shanghai, China; c Department of Dermatology, Huashan Hospital, Fudan University, Shanghai, China; d Department of Dermatology, Eberhard Karls University Tübingen, Tübingen, Germany; e Women's College Research Institute, Women's College Hospital, Toronto, Ontario, Canada; f Division of Dermatology, Department of Medicine, University of Toronto, Toronto, Ontario, Canada; g Nirvan Skin Clinic, Vadodara, India; h Institute and Comprehensive Center for Inflammation Medicine, University of Lübeck, Lübeck, Germany; i Department of Dermatology, Xijing Hospital, Fourth Military Medical University, Xi’an, China; j Department of Dermatology, Xiangya Hospital, Central South University, Changsha, China; k Department of Dermatology, China-Japan Union Hospital of Jilin University, Changchun, China; l Department of Epidemiology and Biostatistics, School of Public Health, TaiKang Center for Life and Medical Sciences, Wuhan University, Wuhan, China; m Shanghai Institute of Immunology, Translational Medicine Center, Shanghai General Hospital, Key Laboratory of Cell Differentiation and Apoptosis of Chinese Ministry of Education, Shanghai Jiao Tong University School of Medicine, Shanghai, China; n Department of Dermatology, Peking University People's Hospital, Beijing, China; o Department of Dermatology, Hainan Provincial Hospital of Skin Disease, Haikou, China; p Department of Dermatology, Zhongnan Hospital of Wuhan University, Wuhan, China; q Department of Dermatology, Shanghai Skin Disease Hospital, Tongji University School of Medicine, Shanghai, China; r Institute of Psoriasis, Tongji University School of Medicine, Shanghai, China; s Cosmetic Laser Dermatology, A Platinum Dermatology Partners Company, San Diego, California; t Department of Dermatology, The Third Affiliated Hospital of Chongqing Medical University, Chongqing, China; u Department of Dermatology, International Mongolian Hospital of Inner Mongolia, Hohhot, China; v Department of Dermatology, Second Affiliated Hospital, Zhejiang University School of Medicine, Hangzhou, China; w Department of Dermatology, Xiamen Chang Gung Hospital, Xiamen, China; x College of Medicine, Chang Gung University, Taoyuan, Taiwan; y Department of Dermatology, First Affiliated Hospital, School of Medicine, Shihezi University, Shihezi, China; z Department of Dermatology, Second Xiangya Hospital, Central South University, Changsha, China; aa Department of Dermatology, General Hospital of Ningxia Medical University, Yinchuan, China; ab Department of Dermatology, The First Affiliated Hospital of Chongqing Medical University, Chongqing, China; ac Hubei Key Laboratory of Bioinorganic Chemistry and Materia Medica, Wuhan, China; ad Hubei Engineering Research Center for Biomaterials and Medical Protective Materials, Wuhan, China; ae School of Chemistry and Chemical Engineering, Huazhong University of Science and Technology, Wuhan, China; af Department of Dermatology, China-Japan Friendship Hospital, Beijing, China; ag Department of Dermatology, Beijing Children’s Hospital, Capital Medical University, National Center for Children's Health, Beijing, China; ah Department of Epidemiology and Health Statistics, School of Public Health, Tongji Medical College, Huazhong University of Science and Technology, Wuhan, China; ai Institute of Dermatology, Chinese Academy of Medical Sciences and Peking Union Medical College, Nanjing, China; aj Department of Dermatology, The Central Hospital of Wuhan, Tongji Medical College, Huazhong University of Science and Technology, Wuhan, China; ak Department of Dermatology, The First Hospital of China Medical University, Shenyang, China

**Keywords:** evidence-based medicine, fractional laser, GRADE, microneedling, modified Delphi method, transdermal drug delivery

## Abstract

**Background:**

Transdermal drug delivery technologies (TDDTs) enhance drug absorption and therapeutic outcomes in dermatology, yet standardized, evidence-based recommendations for their clinical application remain lacking.

**Objective:**

To provide expert consensus recommendations on the clinical application of TDDTs, with a focus on fractional lasers and microneedles.

**Methods:**

A multidisciplinary panel of 25 experts systematically reviewed current literature and clinical practices. The consensus was developed using the Grading of Recommendations Assessment, Development and Evaluation framework to assess evidence quality and recommendation strength and the modified Delphi method to achieve expert agreement.

**Results:**

Evidence-based recommendations were formulated for the use of TDDTs in a variety of dermatological conditions, including cutaneous malignancies and precancerous lesions, viral warts, inflammatory and autoimmune skin diseases, and cosmetic indications.

**Conclusion:**

Fractional lasers and microneedles are valuable transdermal delivery tools for dermatological therapy. This expert consensus provides practical, evidence-based guidance to support their safe and effective clinical use.

## Highlights

• This consensus summarizes evidence on transdermal drug delivery in dermatology.
• Fractional lasers and microneedles have the strongest clinical evidence.
• Evidence was assessed via GRADE and refined through a modified Delphi process.
• Disease-specific recommendations are provided to inform the clinical use of TDDTs.

**Capsule Summary**

• This consensus synthesizes current evidence on transdermal drug delivery technologies in dermatology, with a focus on fractional lasers and microneedles.
• Evidence-based recommendations are presented to guide optimal transdermal delivery technology selection in dermatologic therapy, aiming to standardize practice and improve clinical outcomes.

## Introduction

Transdermal drug delivery (TDD) allows therapeutic agents to cross the skin barrier and exert local or systemic effects.^1^ Conventional transdermal systems, including patches and topical formulations, are limited by the barrier function of the stratum corneum.^2^ Transdermal drug delivery technologies (TDDTs), particularly fractional lasers and microneedles, create controlled microchannels that may enhance drug penetration.^3^, ^4^, ^5^ Although a clinical practice guideline for laser-assisted drug delivery has been published, disease-specific evidence-based recommendations in dermatology remain lacking.^5^

To support the standardized and evidence-based application of TDDTs, the consensus working group invited experts from 5 countries to conduct a comprehensive literature review and structured expert evaluation. Using the Grading of Recommendations Assessment, Development and Evaluation (GRADE)^6^ framework to assess evidence quality and a modified Delphi method^7^ to achieve agreement, this consensus emphasizes fractional lasers and microneedles, which currently hold the strongest and most consistent clinical evidence among TDDTs.^3^, ^4^, ^5^ This document provides practical, disease-specific recommendations for dermatologists, nurses, technicians, educators, and researchers on selecting appropriate technologies and applying them safely and effectively in routine clinical practice.

## Methods

### Evidence retrieval

**Fig 1:**
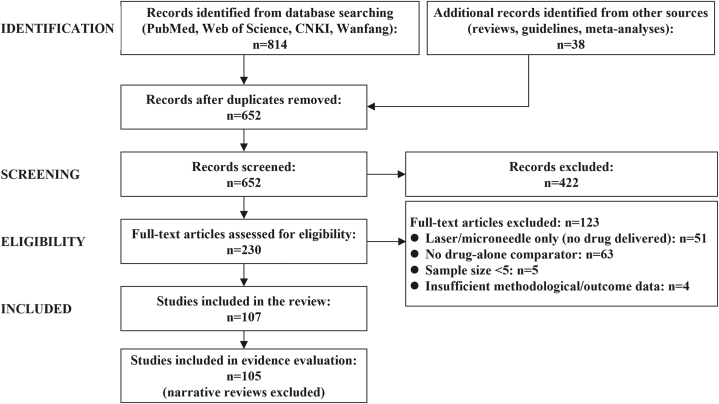
PRISMA 2020 flow diagram for literature screening and study selection. The diagram summarizes the evidence identification and selection process for studies evaluating fractional laser-assisted and microneedle-assisted transdermal drug delivery in dermatology. A total of 652 records were screened, with 422 excluded during title/abstract review. Full-text assessment was performed for 230 articles, of which 123 were excluded for predefined reasons (laser/microneedle monotherapy without drug delivery, lack of a drug-alone comparator, small sample size, or insufficient methodological/outcome data). 107 studies were included in the review; 2 narrative reviews were excluded from evidence grading, resulting in 105 studies included in the evidence evaluation. *PRISMA*, Preferred Reporting Items for Systematic Reviews and Meta-Analyses.

Systematic searches were conducted in PubMed, Web of Science, CNKI, and Wanfang using terms combining dermatologic diseases with TDDTs, supplemented by snowball searches of reviews, guidelines, and meta-analyses. The Preferred Reporting Items for Systematic Reviews and Meta-Analyses flow diagram is shown in Fig 1. Evidence quality and recommendation strength were graded using the GRADE approach. Five drafting members independently assessed the evidence, with disagreements resolved in meetings moderated by evidence-based medicine experts.

### Formation of consensus statements

This consensus was developed by a multidisciplinary panel of 25 experts from 5 countries. Draft statements based on the literature review and expert input were refined through a modified Delphi process involving 2 questionnaire rounds and 2 online meetings. Response rates were 96% (24/25) in round 1 and 88% (22/25) in round 2. Consensus was defined a priori as ≥85% agreement. Further details are provided in the Supplementary Information, available via Mendeley at https://data.mendeley.com/datasets/mvwmr4fm8r/2.

## Results

The panel developed 14 consensus statements. Table I summarizes the evidence levels and recommendation strengths for fractional lasers and microneedles as TDD techniques across dermatological conditions.

**Table I:** Evidence levels and recommendation strengths of fractional lasers and microneedles for transdermal drug delivery across dermatologic diseases

Indications	Drug	Fractional laser		Microneedle	
		Evidence	Recommendation	Evidence	Recommendation
AK	ALA/MAL	High	Strong	Moderate	Strong
BD (inoperable)	MAL	Moderate	Weak	-	-
Noninvasive and microinvasive SCC (inoperable)	MAL	Moderate	Weak	-	-
Low-risk nodular BCC (inoperable)	MAL	Moderate	Weak	-	-
Plantar warts	5-FU	-	-	Moderate	Strong
	Bleomycin	-	-	Moderate	Strong
Facial flat warts	ALA	Moderate	Strong	-	-
AA	Topical corticosteroid	High	Strong	High	Strong
AGA	Minoxidil	Moderate	Strong	High	Strong
Melasma	Tranexamic acid, vitamin C	-	-	Moderate	Strong
Vitiligo	Tacrolimus	-	-	Moderate	Weak
Hand eczema	Halometasone	Moderate	Weak	-	-

*5-FU*, 5-fluorouracil; *AA*, alopecia areata; *AGA*, androgenetic alopecia; *AK*, actinic keratosis; *ALA*, 5-aminolevulinic acid; *BCC*, basal cell carcinoma; *BD*, Bowen disease; *MAL*, methyl aminolevulinate; *SCC*, squamous cell carcinoma.

### Actinic keratosis

Actinic keratosis (AK) is a common ultraviolet-induced keratinocytic lesion and a precursor of cutaneous squamous cell carcinoma (SCC).^8^ TDDTs may be considered when photodynamic therapy (PDT) or topical therapy is used, especially in hyperkeratotic lesions.

#### Statement 1

Fractional laser is recommended for transdermal delivery of photosensitizers in the PDT of AK (evidence level: high; recommendation strength: strong).

Multiple studies have reported higher clearance rates with ablative fractional laser-assisted photodynamic therapy (AFL-PDT) compared with conventional PDT for actinic keratosis.^9^, ^10^, ^11^, ^12^, ^13^, ^14^, ^15^, ^16^, ^17^ A meta-analysis of 4 randomized controlled trials (RCTs) showed that AFL-PDT was associated with higher clearance rates than PDT alone (risk ratio: 1.33, 95% confidence interval: 1.24-1.42, *P* < .01), with no significant differences in pain intensity. However, the included studies showed a high risk of bias.^9^ One RCT reported higher overall complete response rates with AFL-PDT compared to conventional PDT (86.9% vs 61.2%), with the greatest benefit observed in Olsen grade III lesions.^10^ AFL-PDT also showed lower 12-month recurrence rates than conventional PDT (9.7% vs 26.6%) and acceptable safety profiles, with mild and tolerable side effects such as erythema and hyperpigmentation.^10^, ^11^, ^12^ Studies further suggested that AFL-PDT could reduce the incubation time of photosensitizers,^13^ although maintaining standard incubation times may yield superior outcomes.^12^^,^^14^ Regarding laser parameters, lesions with greater thickness or hyperkeratosis (Olsen grade III) may benefit from deeper ablation (eg, 500 μm), whereas thinner lesions (Olsen grade I/II) showed similar outcomes regardless of depth.^15^

#### Statement 2

Microneedle is recommended for transdermal delivery of photosensitizers in the PDT of AK (evidence level: moderate; recommendation strength: strong).

Several small-scale RCTs have reported that microneedle-assisted photodynamic therapy (MN-PDT) is associated with higher clearance rates compared with PDT alone, with acceptable safety and tolerability.^18^, ^19^, ^20^, ^21^, ^22^ Small studies also suggest shorter photosensitizer incubation times with MN-PDT.^22^^,^^23^ Plum-blossom needle, a traditional Chinese medicine therapy, also demonstrated higher clearance rates compared to conventional PDT in a retrospective study.^24^

Two small studies investigated MN-assisted delivery of 5-fluorouracil (5-FU) and imiquimod. Low-concentration 5-FU (0.5% to 5%) after MN pretreatment showed faster onset and fewer side effects without significant efficacy differences.^25^ AFL-assisted imiquimod treatment appeared to improve clearance rates, although without statistical confirmation.^26^

Other TDDTs for PDT in AK include the use of topical keratolytics (eg, salicylic acid, urea),^27^ curettage,^27^^,^^28^ microdermabrasion,^29^ and iontophoresis.^30^ While some studies suggested these methods could enhance PDT efficacy, they were limited by small sample sizes and low-quality evidence.

### Bowen’s disease

Bowen’s disease (BD) is SCC in situ.^31^ TDDTs may be considered when surgery is not preferred and PDT is used.

#### Statement 3

Fractional laser is recommended for transdermal delivery of photosensitizers in the PDT of inoperable noninvasive BD (evidence level: moderate; recommendation strength: weak).

A research team conducted 2 RCTs on lower extremity BD lesions and reported that AFL-assisted methyl aminolevulinate PDT was associated with higher complete response rates at 3 months (93.8% vs 73.1%) and 12 months (84.78% vs 44.74%), and significantly reduced 5-year recurrence rates (9.3% vs 41.38%) compared to methyl aminolevulinate PDT.^32^^,^^33^

A separate small-scale study explored the efficacy of MN-PDT in 3 BD patients who previously had suboptimal responses to curettage and PDT. The results showed all patients achieved complete remission with no recurrence at 3 months, although the evidence level remains very low due to the limited sample size.^34^

### Squamous cell carcinoma

Cutaneous SCC is usually treated surgically.^35^ TDDTs may be considered in selected inoperable noninvasive or microinvasive lesions treated with topical therapy or PDT.

#### Statement 4

Fractional laser is recommended for transdermal delivery of photosensitizers in the PDT of inoperable noninvasive and microinvasive SCC (evidence level: moderate; recommendation strength: weak).

For Clark level II microinvasive SCC (<1 mm depth without perineural or lymphovascular involvement), an RCT showed that AFL-PDT achieved higher 3-month complete response rates (84.2% vs 52.4%) and lower 24-month recurrence rates (12.5% vs 63.6%), with similar cosmetic results, adverse events, and pain to conventional PDT.^36^ Two case series of fractional laser-assisted 5-FU delivery for SCC in situ reported preliminary efficacy without severe adverse events, although larger RCTs are needed.^37^^,^^38^ For advanced SCC with perineural or lymphovascular invasion, PDT with TDDTs may serve as an adjuvant, while transdermal immunotherapy and novel chemotherapies remain under investigation.

### Basal cell carcinoma

Basal cell carcinoma (BCC) is the most common skin cancer, and surgery is first-line treatment for most lesions.^39^ TDDTs may be considered for selected superficial or thin nodular inoperable lesions treated with topical therapy or PDT.

#### Statement 5

Fractional laser is recommended for transdermal delivery of photosensitizers in the PDT of inoperable low-risk nodular basal cell carcinoma (nBCC) (thickness < 2 mm) (evidence level: moderate; recommendation strength: weak).

Evidence on TDDTs for nBCC remains limited. In a small RCT of thin nBCC (<2 mm), Er:YAG AFL-assisted PDT achieved significantly higher complete response rates at 3 months (84.2% vs 50%) and lower 12-month recurrence rates than conventional PDT (6.3% vs 55.6%), with comparable cosmetic and safety profiles, suggesting its potential role as an alternative for patients ineligible for surgery.^40^ For thicker nBCC, 2 small RCTs found that tumor debulking by curettage or ablative lasers before AFL-PDT improved outcomes versus conventional PDT.^41^^,^^42^ Small studies on fractional laser-assisted topical imiquimod also showed improved efficacy without serious adverse events, although evidence remains very low due to limited sample sizes.^21^ In rare cases where BCC invades deep soft tissue or bone and is inoperable, PDT combined with TDDTs may offer an adjunctive therapeutic option.

### Viral warts

Plantar and facial flat warts are common viral lesions, and plantar warts may be recalcitrant.^43^ TDDTs may be considered when enhanced local delivery or less painful treatment is desired.

#### Statement 6

Microneedle is recommended for the transdermal delivery of 5-FU and bleomycin in the treatment of plantar warts (evidence level: moderate; recommendation strength: strong).

In an RCT of 90 patients, microneedling plus 5-FU achieved a higher complete response rate than intralesional 5-FU (86.7% vs 76.7%), with significantly fewer treatment sessions (*P* = .01), lower pain scores (*P* = .001), and higher patient satisfaction (*P* = .05).^44^ In another RCT of 60 patients, microneedling-assisted bleomycin achieved higher complete resolution rates than intralesional bleomycin (83.3% vs 70%) with fewer sessions and less pain.^45^

#### Statement 7

Fractional laser is recommended for transdermal delivery of photosensitizers in the PDT of refractory facial flat warts (evidence level: moderate; recommendation strength: strong).

For extensive and refractory facial flat warts, a retrospective analysis involving 80 patients with refractory facial flat warts revealed that fractional laser-assisted PDT with 5-aminolevulinic acid significantly improved the overall effectiveness and clearance rate compared to PDT alone (81.68 ± 2.83% vs 68.13 ± 3.97%, *P* = .0068).^46^

### Alopecia areata

Alopecia areata is an autoimmune nonscarring alopecia, and local corticosteroids are commonly used for localized disease.^47^ TDDTs may be considered when response to conventional topical therapy is inadequate.

#### Statement 8

Microneedle is recommended for transdermal delivery of corticosteroids in the treatment of alopecia areata (evidence level: high; recommendation strength: strong).

Multiple RCTs have reported that microneedling followed by topical triamcinolone acetonide application is associated with improved clinical outcomes in patients with alopecia areata.^48^^,^^49^ In a 2025 RCT of 80 patients with mild-to-moderate patchy alopecia areata, microneedle delivery of compound betamethasone achieved efficacy comparable to intralesional injection after 3 months, with similar Severity of Alopecia Tool responses and lower pain scores.^50^

#### Statement 9

Fractional laser is recommended for transdermal delivery of corticosteroids in the treatment of alopecia areata (evidence level: high; recommendation strength: strong).

RCTs have reported that fractional CO_2_ laser–assisted delivery of topical triamcinolone acetonide is associated with improved treatment responses in patients with alopecia areata.^48^^,^^49^ In addition, a meta-analysis showed that fractional laser-assisted topical therapy (including corticosteroids or minoxidil) was associated with higher treatment response rates than topical therapy alone, although laser types and treatment parameters varied across studies.^51^

### Androgenetic alopecia

Androgenetic alopecia (AGA) is a common progressive hair loss disorder, and topical minoxidil is a first-line treatment.^52^ TDDTs may be considered when response to conventional topical therapy is suboptimal.

#### Statement 10

Microneedle is recommended for transdermal delivery of minoxidil in AGA treatment (evidence level: high; recommendation strength: strong).

A meta-analysis of 13 RCTs involving 696 patients evaluated the efficacy and safety of microneedling combined with therapies such as minoxidil, finasteride, or platelet-rich plasma. The study found that combination therapies were associated with significant improvements in hair density and diameter compared to monotherapies. Adverse events such as scalp pain and mild erythema were mild and self-limiting, with no significant differences in safety profiles between combination and monotherapy approaches.^53^ Among these, the evidence was strongest for microneedling combined with minoxidil, which consistently improved hair density and, in some studies, hair shaft diameter versus minoxidil alone.^54^, ^55^, ^56^ In one randomized evaluator-blinded study, weekly microneedling plus minoxidil increased hair count more than minoxidil alone at 12 weeks (91.4 vs 22.2 hairs/cm^2^; *P* = .039), and more patients achieved >50% improvement (82% vs 4.5%).^54^

#### Statement 11

Fractional laser is recommended for transdermal delivery of minoxidil in AGA treatment (evidence level: moderate; recommendation strength: strong).

In 2 RCTs of 30 patients each, fractional laser-assisted minoxidil improved hair density and dermoscopic outcomes; one study showed better results at week 24 (all *P* < .05), whereas the other found a higher dermoscopy score despite no significant difference in photographic assessment (*P* = .016 and *P* = .13, respectively).^57^^,^^58^

### Melasma

Melasma is a chronic relapsing pigmentary disorder mainly treated with topical agents. TDDTs may be considered when improved penetration of topical agents is desired.^59^

#### Statement 12

Microneedle is recommended for the transdermal delivery of topical agents in the treatment of melasma (evidence level: moderate; recommendation strength: strong).

A meta-analysis of 12 studies including 459 participants showed that microneedling-assisted topical therapy significantly reduced melasma severity, with a large effect beyond 8 weeks, the greatest effect at 12 weeks, and no severe adverse events. However, variations in microneedling parameters and drug formulations highlight the need for further research to optimize treatment protocols.^60^

Emerging laser-assisted evidence has also been reported in melasma. In a 2025 randomized split-face trial, nonablative fractional laser plus topical tranexamic acid achieved better PGA scores at week 12 (*P* < .001), although the between-group difference was not maintained during follow-up.^61^

### Vitiligo

Vitiligo is an acquired depigmenting disorder.^62^ TDDTs may be considered for localized stable or recalcitrant lesions treated with topical immunomodulators.

#### Statement 13

Microneedle may be considered for transdermal delivery of tacrolimus in recalcitrant vitiligo lesions (evidence level: moderate; recommendation strength: weak).

In an RCT of 90 patients with localized stable vitiligo, microneedling plus 0.1% tacrolimus achieved a higher overall improvement rate (76.6%) and more excellent repigmentation (66.6% vs 33.3%; *P* = .03) than either treatment alone, with mild adverse events.^63^

Several recent reviews have summarized the role of laser technologies in vitiligo management.^64^, ^65^, ^66^, ^67^ The 308-nm excimer and fractional lasers are recognized as stand-alone therapies, with fractional lasers being explored for their potential to facilitate TDD. In a multicenter prospective study (*n* = 289), ablative fractional CO_2_ laser pretreatment combined with topical betamethasone and narrowband ultraviolet B was associated with higher repigmentation rates in acral vitiligo than betamethasone alone, without severe adverse events; however, the independent contribution of the fractional laser could not be determined due to the absence of an appropriate control arm, and the study was therefore not incorporated into the consensus.^67^

### Hand eczema

Chronic hand eczema is a common inflammatory skin disorder, and topical corticosteroids remain first-line therapy.^68^ TDDTs may be considered in recalcitrant or hyperkeratotic disease.

#### Statement 14

Fractional laser may be considered for transdermal delivery of topical corticosteroids to treat recalcitrant moderate-to-severe hand eczema (evidence level: moderate; recommendation strength: weak).

In a randomized trial of 67 patients with moderate-to-severe chronic hand eczema, fractional laser-assisted halometasone achieved a higher week-12 treatment success rate than halometasone alone (62.1% vs 27.6%) and a lower relapse rate at week 24 (11.1% vs 50.0%).^69^

## Conclusion and future perspectives

Among currently available transdermal delivery modalities, fractional lasers and microneedling have the most substantial and relatively consistent clinical evidence to support disease-specific recommendations. Evidence is strongest in AK and AGA, while applications in melasma, BCC, SCC in situ, viral warts, vitiligo, alopecia areata, and hand eczema appear promising but require larger controlled studies.

Future research should standardize treatment parameters, clarify pharmacokinetics, compare delivery modalities directly, and expand long-term safety data. Emerging device-drug platforms, including dissolvable microneedles,^70^, ^71^, ^72^, ^73^ may further broaden the therapeutic role of TDDTs in dermatology.

## Conflicts of interest

Dr Goldman reports serving as an investigator and/or consultant for Biofrontera, Accure, Allergan, Galderma, Lumenis, Solta, SkinMedica, and SkinCeuticals. Dr Piguet reports receiving research grants from AbbVie, Bausch Health, Celgene, Lilly, Incyte, Janssen, LEO Pharma, L’Oréal, Novartis, Organon, Pfizer, Sandoz, Amgen, and Boehringer Ingelheim and honoraria for speaking from Sanofi China. Drs Zhu, Li, Wang, Xiang, Röcken, Verma, Thaçi, Li, Chen, Zhu, Miao, Wang, Zhou, Ding, Xie, Shi, Hao, Mu, Man, Chung, Wang, Xiao, Yu, Chen, Zhu, Cui, Ma, Yin, Bu, Yang, Li, Gao, and Tao have no conflicts of interest to declare.
